# Biliary Calculi, Obliteration of the Biliary Ducts, Abscess of Liver, &c.

**Published:** 1853-05

**Authors:** Ira. E. Oatman

**Affiliations:** Sacramento City, California


ART. III.—*Biliary Calculi, Obliteration of the Biliary Ducts--Abscess of the Liver, &c.* By IRA. E. OATMAN M. D., of Sacramento City, California

ON the 22d of Nov. 1852, I was called to treat Mr. C., attorney-at-law, for an aching pain in that part of the right hypochondriac region occupied by the gall bladder. He was about thirty years old; and of a nervous-bilious temperament. Circulation natural.

He was attacked while riding on horseback, on a professional tour in the mines, from whence he was compelled to return to this city in the stage.

He was treated with cups and mercurial cathartics, followed by sulph. of quinia and sulph. morph., and counter irritation, with only temporary relief. Counsel being called, the Hydrarg. Chlorid. mit. and Morph. Sulph. were given in larger doses, but with the same results. The treatment then employed, was Potassii Iodidum, tonics and anodynes, with beef tea, &c., for nourishment, till his death, which occurred on the 22d, of December. The epigastric and hypochondriac regions gradually enlarged, and became tender upon pressure. The brow was knit all the time; the urine deposited a lithorage like sediment, and the stools were deficient in bile. He had anorexia towards the last, and became extremely emaciated. He did not become jaundiced until a few days before his death. Post mortem 30 hours after death, in presence of other medical gentleman.

The liver, enlarged to near twice its natural size, was firmly adhered over most of its surface, to the diaphragm and the contiguous viscera.

The gall bladder, reduced in size, contained a small quantity of viscid, muco-gelatinous fluid, nearly transparent, and two biliary calculi—one of which was nearly an inch in length. It was slightly loblulated, and of a dark olive brown color. It was friable when dried. The biliary ducts were entirely obliterated—they were reduced to small, firm, white chords.

The liver contained two large abscesses, one of which occupied most of the right lobe, and was pointing towards the right side.—It contained about a quart of yellow purulent fluid, part of which was thin, and the remainder of the consistence of cream.

The other contained about four oz. of the same kind of fluid. It was situated beneath the epigastric region, and extended into the left lobe. It pointed towards the pericardium. The pleural surfaces of the diaphragm and the right lung were firmly adhered.—The apices of both lungs were occupied by tubercles, some of which were mature. The hour having arrived for the funeral, no further examinations were made.

The pathology of this case probably was, that the exercise on horseback forced these calculi into the cervix of the cyst, where they were firmly impacted, the smaller one occupying the original orifice of the cystic duct; and that the obliteration of the ducts, the abscesses, &c., were consecutive.

Sacramento, March 30, 1853.

